# Finding Solutions to Addressing Inequalities in Dementia Diagnosis and Care: Recommendations From a Country‐Wide Consultation

**DOI:** 10.1002/gps.70198

**Published:** 2026-02-26

**Authors:** Clarissa Giebel, Marie Poole, Catherine Talbot, Neil Chadborn, Nadia Brookes, Kritika Samsi, Paul Clarkson, Jacqui Cannon, Mark Gabbay, Kerry Hanna, Aravind Komuravelli, Deborah Rozansky, Hilary Tetlow, Madeleine Walpert, Rosie Whittington, Emma Williams, Louise Robinson

**Affiliations:** ^1^ Department of Primary Care & Mental Health University of Liverpool Liverpool UK; ^2^ NIHR Applied Research Collaboration North West Coast Liverpool UK; ^3^ Population Health Sciences Institute Newcastle University Newcastle upon Tyne UK; ^4^ NIHR Applied Research Collaboration North East and North Cumbria Newcastle upon Tyne UK; ^5^ Department of Psychology Bournemouth University Bournemouth UK; ^6^ School of Medicine University of Nottingham Nottingham UK; ^7^ NIHR Applied Research Collaboration East Midlands Nottingham UK; ^8^ Centre for Health Services Studies University of Kent Canterbury UK; ^9^ Health and Social Care Workforce Research Unit King's College London London UK; ^10^ Social Care and Society Division of Nursing Midwifery and Social Work University of Manchester Manchester UK; ^11^ Lewy Body Society Wigan UK; ^12^ School of Allied Health Professions and Nursing University of Liverpool Liverpool UK; ^13^ Mersey Care NHS Trust Liverpool UK; ^14^ Social Care Institute for Excellence London UK; ^15^ Dementia UK London UK; ^16^ Me2U Centre Liverpool UK

## Abstract

**Background:**

Accessing a diagnosis and receiving adequate care and support for dementia can often be subject to various inequalities. Personal‐, community‐, and infrastructure‐level factors can contribute to and often intersect in causing unequal health and care outcomes. With a paucity of evidence to inform solutions for dementia inequalities, the aim of this public consultation exercise was to explore potential solutions to inequalities in dementia diagnosis and care with different dementia stakeholders.

**Methods:**

Utilising a future workshop approach, we conducted 11 in‐person and remote consultation workshops to discuss experienced barriers of accessing diagnosis and care; discuss an ideal‐world scenario where no barriers exist; and solutions to reach more equitable dementia diagnosis and care with people with dementia, unpaid carers, health and social care professionals, and third sector representatives. Discussions were synthesised by the research team and one public consultation group and mapped against the Dementia Inequalities model.

**Results:**

A total of 131 different stakeholders in dementia attended 11 workshops across England. Solutions were identified across three layers of inequalities, with the majority of solutions proposed on a community and infrastructure level. Examples included link workers, a social care career pathway, Community Champions, adequate home equipment, and digital training. Some solutions require Governmental input, such as creating career pathways in the social care workforce, similar to the NHS, to train and maintain good paid carers, as well as a cross‐UK national dementia strategy raising the priority of dementia and required changes.

**Conclusions:**

Dementia inequalities could be addressed via diverse and holistic approaches. With limited evidence to date on the impact of some of the proposed solutions, future research needs to build on these recommendations and design and test suitable interventions.

## Key Points

- People with dementia and their carers experience a myriad of inequalities, which can be address on a personal‐, community‐, and infrastructure level.
- Across 11 workshops, many solutions to addressing inequalities were identified, including link workers, a social care career pathway, Community Champions, adequate home equipment, and digital training.
- Inequalities need to be address jointly and require a multitude of interventions and strategies to support people with dementia and their carers to access diagnosis and care more equitably.

## 1 Introduction

With nearly one million people living with dementia in the UK (OHE, 2024), not everyone receives a diagnosis or can access the care and support they need and are entitled to. Many people with dementia and their unpaid carers experience multiple intersecting inequalities to receiving a correct and timely diagnosis, and adequate care in the community [1, 2, 3]. Whilst over 100 inequalities in dementia care have been identified in terms of health and social care provision [4], there is an urgent need to co‐develop solutions to these with key stakeholders, including people with dementia, their families and service commissioners and providers.

First categorised into a rainbow of inequalities by Dahlgren and Whitehead [5], health inequalities for the general population have been grouped into individual lifestyle factors, including age and gender; social and community networks factors; and general socio‐economic, cultural and environmental factors, including work environment, unemployment, housing, and health care services. Whilst some of these factors remain the same for the growing population of people living with dementia and their unpaid carers, there are additional specific inequalities faced by this population which are important to be highlighted. Specifically, via consultation processes, the original rainbow model of inequalities [5] has since been adapted into the Dementia Inequalities Model [6]. Building on the previously identified three layers of inequalities, dementia inequalities in diagnosis and/or care can be mapped into individual‐level factors, including age, gender, ethnicity, education, socio‐economic background, and dementia subtype; social and community networks factors, including availability of a carer, living situation, and stigma; as well as society and infrastructure‐level factors, including lack of available services and adequate and timely information provision, lack of workforce knowledge, and failing health and social care system integration [1, 2, 3].

Whilst inequalities in diagnosis and care, and their contributing factors, have received ample attention in recent years, less focus has been placed on directly addressing individual or multiple inequalities. To date, no study has systematically explored potential solutions for various factors of inequalities. Therefore, public consultations can act as a next step towards identifying possible solutions, to inform research and implementation.

Therefore, the aim of this public involvement exercise as part of the Equadem Network Plus was to co‐produce solutions to inequalities in dementia diagnosis and care. With an abundance of evidence on the inequalities and intersection of inequalities which people with dementia and unpaid carers can face in relation to accessing diagnosis and using post‐diagnostic care, now is the time to find solutions to create equity in access and use.

## 2 Methods

### 2.1 Equadem Network Plus

Equadem Network Plus is a funded network designed to bring together diverse stakeholders in dementia to jointly find solutions to dementia inequalities, including researchers, health and social care professionals, third‐sector workers, and people with lived experience. The network holds regular in‐person and remote events to facilitate networking, learning, and skill‐building, and is located across seven main sites in England, linked to Universities (Liverpool, Newcastle, Manchester, Bournemouth, Nottingham, Kent, and London). Since Year 1, one University has left the network (Kent).

### 2.2 Workshop Attendees and Recruitment

People living with dementia, unpaid carers, health and social care professionals, third sector representatives, interested members of the public (‘community advocates or champions’), and commissioners were eligible to participate in the consultation workshops. Participants had to be aged 18+. Potential attendees were informed about the workshops via the Equadem Network Plus mailing list, sharing in local support groups, NHS Trusts and social care providers as well as social media and the NIHR Applied Research Collaboration (ARC) networks. We emailed care providers directly with the workshop flyer, adapted for each location with date and venue. Interested participants could email the workshop leader and thus be signed up for the workshop.

### 2.3 Consultation Workshops

We held workshops in seven sites linked to the UK Universities that are part of the core team of the Equadem Network Plus. Workshops were held at University premises or local community groups, or online. Workshops either involved a mix of stakeholders, including people with dementia, unpaid carers, health and social care professionals, and third sector representatives, or had a more specific focus, such as on the social care workforce, or different personal characteristics such as ethnic background or gender and sexuality.

At each workshop, attendees sat at group tables, which were facilitated by core team members of Equadem Network Plus. This included three carers as part of the research team who were given specific training and support. Each workshop lasted a maximum of 2 hours and started with a brief talk from a member of the research team about existing evidence on dementia inequalities. Subsequently, each group was encouraged to reflect upon their own experiences or ideas of how equity in dementia diagnosis and care can/could be achieved. These consultations adopted a ‘future workshop’ approach [7], which comprised three phases: Phase 1, critiquing the current situation; Phase 2, envisioning the future; and Phase 3, implementable solutions or moving from the present to the imagined future. This approach can foster empowerment, collaboration, and innovative thinking [8]. However, future workshops can produce solutions which are not yet feasible; for example, Haimson et al. [9] found participants often proposed technological solutions beyond current possibilities. This allows for innovative thinking though that goes beyond the currently known.

Participants began by mapping out the challenges encountered by people with dementia and carers, using post‐it notes to then be categorised into broader themes. Next, facilitators guided participants through a scenario‐building exercise, envisioning a future society where the discussed inequalities have been substantially reduced or eradicated. During this phase, participants were encouraged to consider imaginative, creative, and innovative solutions. This activity was complemented by diagramming, in which participants recorded any specific actions and initiatives, as well as the practicalities of imagined solutions on post‐it notes which were mapped against the Dementia Inequalities model layers of inequalities (Individual; Social and Community Networks; Society and Infrastructure) on A3 paper. The workshop discussions finished with a collaborative action planning session, during which attendees identified tangible steps and strategies for implementing the proposed solutions within communities. Attendees were reimbursed for their time with a shopping voucher.

One workshop was held remotely with a total of three people. Due to the small number, no breakout rooms were required. The same structure of the workshop was applied. A Padlet board (with virtual post it notes) was created for attendees, but they did not feel comfortable using it, so we had a structured discussion around those phases. The facilitator took notes on action points and at the end of each phase repeated these back to attendees to ensure everything was captured correctly.

### 2.4 Synthesis of Discussion Points

After each workshop, the workshop facilitators collated all notes and shared them with the team. All workshop facilitator leads and one public advisor met to generate a summary of key solutions identified and whether there was cross‐over across workshops. For this, each workshop lead presented key solutions identified at their respective workshop(s), with each subsequent workshop facilitator presenting stating whether the same or additional or different solutions were discussed. These highlights were shared with the Liverpool‐based public consultation group to discuss, comprising of seven carers and one person with dementia having attended the meeting. Attendees discussed how some of these solutions might have helped or would help them in their own journeys, stressing that the identified solutions would have to be implemented in parallel, as no single solution can address all barriers facing people with dementia and carers. No solution was identified as more important than another.

### 2.5 Public Involvement in the Project Team

Two unpaid carers who were public advisors (JC, HT), and one unpaid carer who is the programme manager of Equadem Network Plus (EW), co‐facilitated one workshop in Liverpool by leading group table discussions, taking notes, and feeding back to the wider workshop attendees. All three were supported in co‐facilitating the workshops where required, and both public advisors were reimbursed for their time. One public advisor and the programme manager were involved in meeting to synthesise the discussions of all workshops, and all helped to finalise the highlighted solutions.

One of two Equadem public consultation groups with eight attendees was also consulted with the summary of key highlighted solution ideas resulting from the core team discussion meeting. The group comprised seven unpaid carers, two of whom were working in the NHS, and one person with young‐onset dementia. Attendees were reimbursed for their time.

### 2.6 Ethics

This was a public consultation exercise without any data collection, and thus did not require ethical approval. To ensure publication eligibility, we submitted this as an ethics application to the University of Liverpool ethics committee, who confirmed it did not require ethical approval [Ref: 14381].

## 3 Results

### 3.1 Overview of Workshop Attendees

A total of 131 people attended 11 workshops across seven sites in England. Table 1 shows details of workshop attendee numbers by background and by location. Forty‐seven percent of workshop attendees (*n* = 61) were unpaid carers, followed by third sector representatives (*n* = 18), social care professionals (*n* = 13), health care professionals (*n* = 12), people with dementia (*n* = 12), 1 commissioner, and others with a wider background in dementia (*n* = 14). Wider background includes community group leaders, including in faith‐based groups. Appendix I includes examples of post it notes from the workshops.

**TABLE 1:** Overview of workshop attendees.

Location	Focus	Number of participants by background							
		PLWD	Unpaid carers	NHS professionals	Social care professional	Third sector	Commissioner	Other	Total
Liverpool	General	3	9	3	1	2	0	3	21
Bournemouth	LGBTQ+ (1:1)	1	0	0	0	0	0	0	1
Bournemouth	Hampshire & dorset	1	21	0	0	0	0	0	22
Bournemouth	LGBTQ+	0	1	1	0	0	0	0	2
Newcastle	SES/rural ‐ family carers and HSCP/VCSE	0	6	0	3	1	0	0	10
Newcastle	SES/rural	0	2	0	0	2	0	0	4
Nottingham	Indian community	0	2	0	0	4	0	2	8
Nottingham	Muslim community	0	3	0	0	4	0	5	12
Manchester	South Asian and BAME communities	0	4	4	0	2	1	0	11
Kent	Mixed group	7	10	4	3	0	0	3	27
London	Workforce	0	3	0	6	3	0	1	13
Total number of participants		12	61	12	13	18	1	14	131

### 3.2 Identified Solutions to Inequalities Mapped Against Dementia Inequalities Model

**FIGURE 1:**
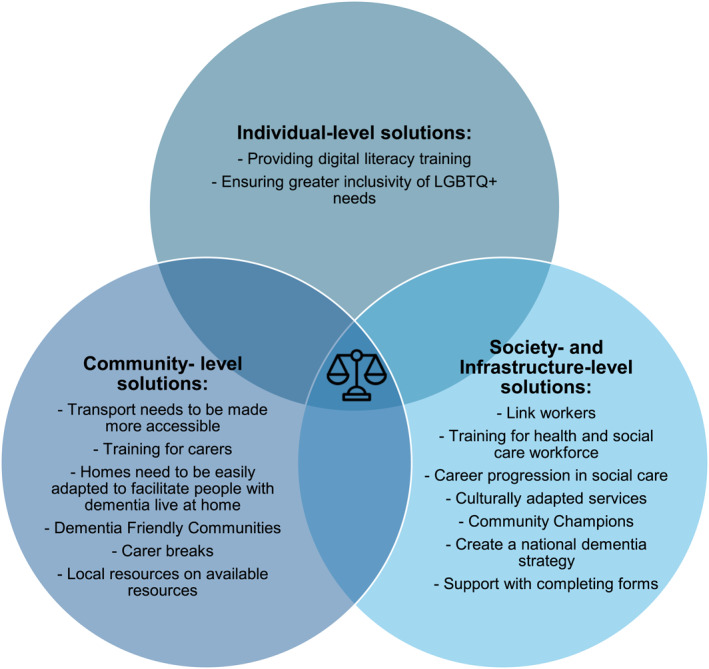
Solutions to inequalities in dementia by each level, and their overlap. Only when solutions on all three levels of inequalities are addressed can a person with dementia reach greater equality in dementia diagnosis and care. Therefore, we used a Venn diagram to show the need for solutions on all levels.

The majority of ideas generated addressed inequalities at a society and infrastructure level or community level, with fewer solutions discussed on an individual level. Attendees highlighted that rather than a single solution, approaches to reduce inequalities need to be implemented in parallel, such as awareness raising activities in the general population as well as improving health and social care integration by improving communication between health and social care professionals. These are examples, with all identified solutions outlined below by inequality level, and also represented in Figure 1. Figure 1 is in the shape of a Venn diagram as only by addressing solutions on each of the three layers can achieve equality in access to diagnosis and care in dementia.

#### 3.2.1 Solutions on an Individual Level

Workshop attendees raised concerns about the digitalisation of health and social care and wanted to see alternatives to digital care and online access points for information. Some attendees suggested that *training for digital skills* could address some accessibility issues related to the digital divide. One further solution, which is linked to the infrastructure level, is that local support groups, day care, home care, and residential care services *need to be culturally appropriate* to enable people with dementia from minority ethnic backgrounds and their carers to access formal care also. This may start with addressing language barriers and other aspects may be tailored to the cultures of the local communities, for example providing halal food for Muslim people and celebrating specific cultural festivities throughout the year. In addition, recognising the diversity of cultures and experiences *within*, as well as across, ethnic groups and the multi‐generational nature of caring by unpaid carers in some groups, such as south Asian carers, was considered important. Sexual and gender minority people affected by dementia discussed similar solutions, including the need for services to actively recognise and *demonstrate inclusivity for LGBTQ+ individuals*, through improved staff training on LGBTQ+ issues, using language that is affirming of diverse identities, better representation in marketing materials, and increasing LGBTQ+‐specific services across the country.

#### 3.2.2 Solutions in Social and Community Networks

##### 3.2.2.1 Living Situation

Considering living situations, emphasis was placed on the problems facing family carers. Possible solutions included the provision of *training for family carers*, highlighting training to encourage better understanding of changes indicative of dementia, and training to manage behaviours which challenge and support communication, as well as digital training. This was mentioned at workshops in Dorset and Hampshire, Liverpool and Newcastle in particular. More broadly, it was suggested that professionals and services need to find ways to *improve communication* with wider family members, highlighting a cross‐layer solution across both community‐level factors and infrastructure factors. Carers need to be valued, trusted and given sufficient time to make decisions, in the context of acknowledging competing demands such as managing families, work and care. In addition, family carers need *breaks within their own home* to reduce carer burnout and support the carer's own health and well‐being, as specifically identified in the Dorset and Hampshire workshop. In terms of the home environment, *homes need to be suitable and accessible for the person as their needs change,* as raised at the Liverpool workshop.

##### 3.2.2.2 Dementia Friendly Communities

Participants discussed multiple broader and intersectional inequalities preventing communities from being dementia friendly. Several practical suggestions were made regarding raising awareness of dementia in local communities through better engagement. These included *intergenerational dementia awareness initiatives* with education in schools and pairing up institutions such as care homes and schools or nurseries to foster longer term connections (mentioned specifically in Liverpool, London, and Newcastle); *games; religious and cultural institutions* and leaders including mosques, temples, mandirs, gurdwaras sharing talks and presentations; and *symbolic gestures* such as removing names from football shirts for high‐profile games, as the Alzheimer's Society has done to showcase that people with dementia experience memory loss.

Using *local resources to raise awareness of dementia* *support* was also considered key across all workshops, including via community centres, local businesses and GP practices, noting the importance of information being current and kept up to date. Both physical and virtual community spaces were considered important for promoting awareness and engagement. These included community centres, public spaces, and local media including television and radio.

*Community events* which take information about dementia into local communities such as dementia workshops, drop‐in centres and mobile hubs (e.g., dementia buses) were proposed by the majority of workshops. In addition, information must take into account low literacy levels and language for some communities where there are high levels of deprivation and multi‐ethnic communities. It was also highlighted that the timing of community social events must be more appropriate for people living with dementia and carers. This involved facilitating events/meetings/groups not necessarily during working hours and not during busy morning and late afternoon times.

Participants at various workshops including Liverpool, Newcastle, and Nottingham suggested that *dementia prevention and risk programmes* could be held at venues such as leisure centres, with education about dementia risk in schools and through charities who have trusted relationships within the community (one example being a football foundation), highlighting links between physical health and prevention.

A *‘neighbourhood approach’* was suggested to help communities to become more dementia friendly, taking advantage of existing initiatives such as Alzheimer's Society Dementia Friends for training; and upskilling local service providers such as shops and employers about dementia. A range of roles were identified which could promote community awareness and engagement. This included established community organisations as *‘brokers’* between the individual and services for example the person at the charity shop who helped with benefits paperwork, or the Tesco community champion. *Community Champions,* who could be located in places such as schools, shops, places of worship, and care settings, were also described by attendees at workshops including in London, Manchester, Nottingham, Dorset and Hampshire, as a possible solution to raising awareness of dementia and championing support and acceptance for people with dementia and their carers in the community, thereby reducing stigma.

##### 3.2.2.3 Transport

It is important to avoid social isolation and to continue to access resources in communities and this requires accessible transport. Community transport is available in some areas but not others, which is particularly an issue for more rural areas. *Transport should be accessible* for people with physical impairments. Driving remains an important aspect of accessible transport for some people, including for people in rural areas, however parking can be difficult, therefore *blue badges* are important. Availability of blue badges should be improved, and these should not need renewing as dementia is irreversible.

##### 3.2.2.4 Network of Dementia Peers

*Peer* *support networks* were considered a helpful solution for finding out what support is available locally, for connecting others experiencing similar issues, and for finding out other information about dementia. One practical suggestion offered was *the use of social media and WhatsApp groups* to keep carers connected in South East Asian communities, which was specifically discussed in Nottingham‐based workshops. In some regions, including Dorset and Hampshire, ways to facilitate access to peer support networks was generally by raising awareness of existing groups, which could be facilitated via WhatsApp group and other means.

#### 3.2.3 Solutions on a Society and Infrastructure Level

Attendees across different workshops identified various possible solutions to address inequalities in dementia diagnosis and care at a wider societal and health and social care infrastructure level, including at the linkage points between diagnosis and moving onto care and support within the community.

From a societal point of view, attendees across all workshops raised the issue of raising public, practitioner, and carer/people with dementia awareness of dementia. Whilst this is primarily covered on the Community level of inequalities, one way to raise awareness from an infrastructure point of view is via the national government by *making dementia a national priority,* including via a national dementia strategy. This could then influence local decision‐makers and also support creating more regional and local dementia plans and strategies, including increasing diagnosis rates and improving access to care. Attendees across workshops also emphasised that securing top‐level commitment from the government is crucial for driving meaningful and lasting solutions across other levels. Targeting improvements on a health and social care infrastructure level, attendees raised various solutions ranging from improved integration between health and social care via a *dedicated link worker*, such as a Dementia Care Navigator, Admiral Nurse, or Dementia Advisor; a regularly updated and easily accessible *directory of available services*; as well as *better* *support with completing necessary forms* (i.e., needs assessment, power of attorney, carers allowance, etc). Attendees highlighted that services as well as forms need to be available *not solely in digital form*, something that has increased particularly since the pandemic but inhibits people with dementia and carers with limited digital skills or access to support. Connecting services better can also be achieved by *improved link up between GPs and third sector organisations*, who provide a great deal of support for people with dementia once diagnosed, and their carers, across the UK. This would generally feed into more *consistent and coordinated* care, as highlighted across all workshops, which could reach out into the community via *Community Champions*, as highlighted by carers and people with dementia in the Dorset and Hampshire workshop. These could be embedded within faith groups also, especially for people from different cultural backgrounds including South Asian ethnicity, as identified in the Manchester‐based workshop. Reducing inequalities at an infrastructure level could also be achieved by taking learnings from more effective health and social care integration or service provision for other conditions and population groups, as raised particularly in the Newcastle‐based workshops.

Focussing on supporting the workforce better was raised with different complementary solutions in mind. At the Liverpool workshop, for example, each of the four group tables raised *more appropriate dementia training for both workforce and unpaid carers*, as a way to ultimately receive better patient care. Attendees at this and other workshops identified their personal experiences of seeing health and social care professionals often lacking adequate knowledge about dementia, dementia subtypes, and required support, which was also the case for unpaid carers attending the workshops. This could be embedded within *creating a social care career pathway with relevant qualifications*, to not only ensure greater knowledge in the social care workforce but also to improve the sector as a more appealing and long‐term employment sector.

## 4 Discussion

A recent national report identified over 100 inequalities in dementia care [4]. This consultation exercise is the first exploration of identifying intersecting solutions to address these different levels and types of inequalities in dementia diagnosis and care, providing a paramount step forward to progress towards creating greater equity in dementia. To date, research has focused on identifying inequalities in dementia diagnosis and care [2, 10], providing a rich background to generate ideas for solutions. With over 130 different stakeholders in dementia involved from various personal, professional, and geographical backgrounds, this consultation study has identified a myriad of potential solutions to create greater equity in diagnosis and care.

Most identified solutions targeted society and infrastructure, or community level causes of inequalities. On the former level, providing link workers from the point of diagnosis to help people with dementia and their carers navigate the dementia journey—from helping with legal documents such as power of attorney or social care needs assessments, to identifying suitable care and support services for both, helping with advanced care planning, and providing a listening ear on a regular basis—was one of the many solutions discussed across the workshops. To date, there is no single such role available in the UK, albeit various roles provide such services, where available. These include non‐clinical roles such as Dementia Care Navigators, link workers or Advisors, and clinical roles such as Admiral Nurses, amongst others. The clinical experience of the latter means such services are often viewed as more costly than using non‐clinically qualified link workers. However, evidence to date suggests that they may be more cost effective in the longer term [11, 12]. The availability of these support systems also varies geographically and can be a postcode lottery as to who receives support. Some national services exist, such as the Admiral Nurse Helpline and Admiral Nurse posts in Nationwide Bank branches. The evidence base for non‐medical link worker roles in dementia is largely from the US [13], where they present a promising model of post‐diagnostic dementia care and support by positively affecting health care utilisation and transitions from the home (i.e., [14, 15]). Research is required to fill this gap and evidence the impact of these roles in UK, and other country and health care system, settings.

Other discussed solutions included improving health and social care professional knowledge of dementia by implementing and improving dementia‐related training, but also by creating a social care career pathway. Concerning existing educational interventions, various educational interventions have been evidenced to improve knowledge, attitudes, empathy, and changes to practice [16, 17, 18]. However, these are one‐off tested interventions, which are not regularly implemented in health or social care practice. Training needs could further be supplemented by establishing a social care career pathway, which currently is missing in the UK. Creating a career pathway for this workforce could also try and address some of the other issues that the workforce and sector at large are facing, including lack of recognition, training, support, and pay, leading to high levels of staff turnover and reduced workforce retention [19].

The health and social care workforce was not the only group that was highlighted to require more knowledge about dementia. There is a need for more general awareness, training and support interventions for people with dementia and carers, as well as the general public, including in community settings. Dementia Champions and Dementia Friendly Communities were mentioned by attendees, both of which have been evidenced to a limited degree to show improvements in awareness about dementia and improving the lives of people with dementia and carers [20, 21, 22]. To overcome the challenges of stigma, teaching children about dementia from an early age is pertinent. One existing educational programme for secondary school children is Dementia Detectives—a one‐hour dementia awareness raising programme for teenagers aged 14–16 [23]. However, this programme has not been evaluated yet for its impact on knowledge. In contrast, a programme called ‘Understanding Dementia: Class in a Bag’ has been shown to improve awareness of dementia in primary school children aged 7–11 and creating empathy for people with dementia [24]. The programme comprises a bag with awareness raising tools, including a storybook, and is made up of three linked activities to improve knowledge about the brain, dementia, and how to help. One further innovative educational tool that significantly improves knowledge about dementia, and associated inequalities, in the general population, and allied health, health care, and psychology undergraduate students is the Dementia Inequalities Game [25, 26]. Whilst a board game that is played for up to 1 hour cannot replace training in the health and social care workforce, it can be an important add‐on to facilitate additional learning in a social environment, as playing a board game in person allows players to talk and share their own experiences of dementia too. Thus, there are innovations to improve knowledge about dementia in the general population, care professionals, and children, which need to be wider implemented to also try and reduce stigma, thus enabling more people with suspected symptoms to seek out help.

To ensure that challenges in dementia diagnosis and care get addressed, including by raising greater awareness in different groups, a national dementia strategy is critical. Since the Prime Minister's Challenge on Dementia 2020, there has been no joined national dementia strategy across all four UK nations. A recent review on existing national dementia strategies, accessible in English or French, in countries that are part of the Organisation of Economic Cooperation and Development, with a particular focus on mentioning inequalities, has shown that the UK is falling behind by not having a current linked up national dementia strategy [27]. Only Scotland has a current National Dementia Strategy [28]. By filling this gap, other solutions may be supported also, such as creating a social care career pathway, embedding better training for the care workforce, children, and general population in workforce, educational, and public health curricula, and by investing more funding into dementia research and care.

Contrary to the community and wider society and infrastructure level of inequalities, fewer solutions were discussed on an individual level. This might reflect a sense that inequalities at this level are too diverse and dependent on the higher levels of more structural and system causes. Those that were highlighted were linked to service suitability (a barrier embedded in the society and infrastructure level), including making post‐diagnostic services such as care homes and day care centres more culturally appropriate with diverse staff with diverse linguistic skills, as well as offering culturally appropriate food. Whilst there is some evidence on how to make care settings more culturally appropriate for different ethnic backgrounds, including for older adults from an Indian heritage [29], there appears to be no evidenced intervention on embedding culturally appropriate care in care settings to date. Focussing on another way to make care more equitably accessible, people with dementia should be supported to be more digitally literate in order to access information and services, whilst simultaneously ensuring that there remains a substantial proportion of in‐person services and access points. When implementing an online peer support group for isolated carers of people with dementia, Banbury et al. [30] found that digital literacy was a challenge and IT support was highly valued, leading to increased confidence and willingness to engage in the group. Despite this, there is currently a lack of research implementing and evaluating digital skills training for people affected by dementia. Additionally, any digital solutions must be supported at a societal and infrastructure level through policies that address digital poverty, including improving internet infrastructure and access to digital devices in all areas. Moreover, websites and digital systems need to be easier to navigate and more user‐friendly. While such initiatives exist for other populations (Digital Poverty Alliance, n.d.), there has been limited focus on how they can be tailored to meet the unique needs of people affected by dementia.

With other solutions raised throughout and across the consultation workshops, this study highlights a multitude of potential paths towards implementing equity in dementia diagnosis and care. Importantly, as also identified in the public consultation group, these solutions should not be considered in isolation, but in parallel, to complement one another. This is because the landscape of inequalities in reaching a timely and correct diagnosis and post‐diagnostic care are too complex to be addressed by a single solution. Creating consistent and coordinated care can be challenging but this consultation exercise has highlighted clear pathways for generating greater equality in access to care, where it is available.

## 5 Limitations

Whilst this consultation exercise benefitted from diverse stakeholder representation from various personal (including young‐onset, LGBTQ+, and South Asian), professional, and third sector backgrounds, as well as from a diverse geographical spread across England, and a large number of attendees, some characteristics were perhaps not captured as much as others. Only 12 of over 130 attendees were living with dementia themselves. This may have limited direct lived experiences, although a lot of unpaid carers participated across the workshops also. It was difficult to recruit people with dementia considering that we did not attend established groups, but facilitated mixed workshops. The workshops included limited representation from migrant populations other than those from a South Asian background. Considering different cultural preferences for care and potential challenges [31], it is important to establish additional solutions to dementia diagnosis and care with lived and care experts from a variety of cultural backgrounds. This may then also provide additional insights into solutions in other countries, which are to be explored separately and currently ongoing in India. Furthermore, our attendees were largely drawn from existing networks and relationships, so seldom involved groups may not have been aware of the meetings or more unsure about participating within unfamiliar settings. However, whilst some groups were not as well represented, we did capture different backgrounds where possible due to our far‐reaching recruitment strategy.

## 6 Conclusions

There are multiple ways in which dementia inequalities could potentially be addressed, based on identified solutions from diverse stakeholders. These include Dementia Champions in the community, Dementia Care Navigators and Admiral Nurses to help navigate the post‐diagnostic journey, as well as improving education of the health and social care workforce about dementia and how to communicate better. Whilst some of these interventions have been minimally evidenced and show early positive impacts on reducing barriers to diagnosis or care, the next step will be to test identified solutions for scaling up and wider implementation, where found to be successful. It will be crucial for research to evidence the cost‐effectiveness of such solutions to influence policy makers and commissioners, who have to look for cost saving options in the NHS and in the already under‐invested social care sector. Measuring for example how link workers may reduce avoidable health care utilisation would be an important next step in research. Given the growing number of dementia cases worldwide, it is important to apply a more positive lens now and shift the focus from the identified challenges, to testing and evidencing solutions to create positive change.

## Funding

This grant (ES/Z502674/1) is funded under the Dementia Network Plus initiative, which is co‐funded by the Economic and Social Research Council (ESRC) and National Institute for Health and Care Research (NIHR), which CG is leading. This research is also supported by the National Institute for Health Research Applied Research Collaboration North West Coast (ARC NWC), NIHR ARC North East and North Cumbria, and NIHR ARC East Midlands. The views expressed in this publication are those of the author(s) and not necessarily those of the National Institute for Health Research or the Department of Health and Social Care.

## Conflicts of Interest

The authors declare no conflicts of interest.

## Data Availability

The data that support the findings of this study are available from the corresponding author upon reasonable request.
